# LaMer growth of iron nanoparticles for magnetic particle imaging

**DOI:** 10.20935/acadmatsci8015

**Published:** 2025-12-08

**Authors:** Aleia G. Williams, Willem Graham, Sydney Henriques, Todd D. Giorgio, Charles E. Johnson, Jacqueline A. Johnson

**Affiliations:** 1 Department of Biomedical Engineering, University of Tennessee Space Institute, Tullahoma, TN, USA.; 2 Department of Biomedical Engineering, Vanderbilt University, Nashville, TN, USA.; 3 Center for Laser Applications, University of Tennessee Space Institute, Tullahoma, TN, USA.

**Keywords:** LaMer growth mechanism, magnetic particle imaging, iron nanoparticle, superparamagnetic, core–shell structure

## Abstract

Magnetic particle imaging (MPI) is a relatively new imaging technique that uses magnetic nanoparticles as tracers to generate a signal. Compared to magnetic resonance imaging, MPI detects the magnetization of nanoparticles directly rather than relying on secondary effects of magnetic resonance relaxation times from protons. Iron oxide nanoparticles with diameters in the 25–30 nm range are among the most commonly used MPI tracers. However, we hypothesize that further improvement in tracers can be achieved by utilizing pure iron nanoparticles. From physics-based models, iron nanoparticles with diameters of at least 20 nm are needed to enhance MPI performance. Previous studies have used thermal decomposition of organic iron precursors to create iron nanoparticles between 5 and 15 nm in diameter, but few studies have successfully created iron nanoparticles of larger sizes. Therefore, we investigated the use of an extended LaMer mechanism to create larger iron nanoparticle sizes for potential MPI applications. Continuous addition of Fe(CO)_5_ as the precursor using a thermal decomposition method was used. Three injection speeds of the iron precursor were tested, which included 100, 50, and 25 μl/min to test if injection rates affected the overall particle growth and size. In addition, three different surfactants, an oleylamine/oleic acid (OAm/OA) mixture, hexadecylamine (HDA), and octadecylamine (ODA), were used in order to test if the surfactants affected the size, morphology, and composition of the nanoparticles. Larger nanoparticle diameters up to 24 and 26 nm were observed at lower injection speeds when HDA and ODA were used, respectively. However, samples using OAm/OA mixtures remained around 15.5 nm at all injection speeds. In addition, OAm/OA samples displayed high magnetic saturation values up to 138 emu/g, but samples using HDA and ODA displayed lower magnetic saturation values up to 46 emu/g and 38 emu/g, respectively.

## 1. Introduction

Magnetic particle imaging (MPI) is a novel, noninvasive imaging technique that was introduced in 2005 by Gleich and Weizenecker [1-4]. MPI is highly competitive in sensitivity, contrast, and spatial and temporal resolution when compared to current clinical techniques such as magnetic resonance imaging (MRI), computed tomography (CT) and positron emission tomography (PET) [5-8]. The use of low-frequency magnetic fields minimizes MPI signal attenuation by anatomical structures or tissue depth, which allows for high-resolution images with excellent contrast [1, 9-13]. Currently, superparamagnetic iron oxide nanoparticles (SPIONs) in the range of 25–30 nm in diameter are the standard material used as magnetic tracers for MPI due to ease of producibility, low toxicity, and good magnetic properties [14-20]. However, we hypothesize that improvements in MPI performance can be achieved by utilizing tracers composed of pure iron instead of iron oxide.

Nanoparticle size and composition are important determinants in the performance of MPI. Theoretically, the signal intensity and resolution for MPI can be improved by increasing the magnetic diameter of the nanoparticle [21, 22]. Therefore, creating optimally sized iron nanoparticles (FeNPs) for MPI applications is the objective of this research. Although many studies have demonstrated the fabrication of iron oxide nanoparticle diameters between 5 and 30 nm, fewer studies have been performed in synthesizing pure FeNPs of similar sizes [23-25]. Synthesis of pure FeNPs above 15 nm in diameter has proven to be a challenge due, in part, to the stronger magnetic properties pure iron possesses when compared to iron oxide. At large sizes, greater magnetic capabilities are observed in FeNPs, which can lead to the flocculation of nanoparticles in solution when improperly stabilized [26]. Dash et al. made iron core–iron oxide shell nanoparticles of three different sizes, 8, 12, and 15 nm, using thermal decomposition of iron pentacarbonyl, and oleylamine and oleic acid as surfactants. They found that 12 nm nanoparticles could be synthesized by adding increased amounts of oleylamine to the reaction. For the 8 nm nanoparticles, smaller heating increments were employed along with higher amounts of oleylamine and oleic acid. Although different sizes of FeNPs were achieved, the maximum size of the nanoparticles was only approximately 15 nm [27].

In order to produce FeNPs with diameters greater than 15 nm, a previous study explored the use of a LaMer growth mechanism [28]. LaMer growth allows particle size control based on the amount of precursor injected over an extended period of fabrication time. The original LaMer mechanism consists of three stages and is shown in Figure 1a. The first stage begins when the precursor is first introduced into the reaction, and the concentration of monomers increases until it reaches a critical supersaturated state. Stage 2 starts when burst nucleation occurs to partially relieve the supersaturation that causes the monomer concentrations to drop below the critical nucleation concentration. Finally, in the third stage, the growth of nuclei occurs by diffusion of the monomer to the particle surface, and the monomer concentrations approach the lower solubility limit (C_s_) [28]. Further growth of the nanoparticles will occur through Ostwald ripening. However, this original LaMer synthesis protocol is susceptible to large size dispersity of the final nanoparticles. Therefore, an extended version was proposed by Vreeland et al., as shown in Figure 1b, where the first and second stages are the same as the original, but the concentration of the monomer decreases in stage 3 [26]. As the concentration of monomers is consumed in stage 3, nanoparticle growth occurs. Due to a continuous addition of the precursor, the production of monomers can be maintained indefinitely. Eventually, the consumption of monomers by the growing nanoparticles will become equal to the rate of production of monomers, and the monomer concentration reaches a steady growth state. A fourth stage is added, which allows a steady state of nanoparticle growth that will, in theory, allow for a wide range of particle sizes while maintaining low size dispersity [26].

This study explored the potential of using the extended LaMer growth mechanism to create FeNPs larger than the ≈15 nm diameter limit of other fabrication approaches. A continuous injection of iron pentacarbonyl was tested at 100, 50, and 25 μl/min injection speeds. In addition, three different surfactants were used at various injection speeds to determine changes in particle size and dispersibility. From this study, we aim to determine the effect that precursor injection rates and surfactants have on FeNP sizes and compositions. In addition, we aim to achieve FeNP sizes of approximately 20 nm for potential MPI applications.

## 2. Materials and methods

### Chemicals:

Iron (0) pentacarbonyl [Fe(CO)_5_, >99.99%], hexadecylamine (HDA 90%), octadecylamine (ODA 90%), 1-octadecene (90%), oleic acid (OA) (90%), oleylamine (OAm) (70%), pentane, diethylether, tetrahydrofuran, chloroform, hexanes, hydrochloric acid (HCl, 37%), and nitric acid (1 M) were purchased from Sigma Aldrich (St. Louis, MO, USA). 1,2-distearoyl-sn-glycero-3-phosphoethanolamine-N-[methoxy-(polyethylene glycol)-2000] (ammonium salt) (DSPE-mPEG) was purchased from Avanti Lipids (Alabaster, AL, USA). Anhydrous ethanol was purchased from Decon Labs (Prussia, PA, USA). Stainless steel 6-inch syringe needles 18-gauge and 3 ml syringes were purchased from Sigma Aldrich (St. Louis, MO, USA).

### FeNP synthesis:

Synthesis of the surfactant, HDA·HCl, was performed 24 h before FeNP synthesis and referenced a procedure from Dash et al. [27]. In an Erlenmeyer flask, 0.488 g of HDA was dissolved in 100 ml of pentane and magnetically stirred. In a separate beaker, 250 μl HCl and 30 ml of diethylether were mixed together. The HCl and diethylether solution was slowly poured into the HDA–pentane solution and mixed at room temperature for 2 min. A white precipitate formed and was gravity-filtered through Whatman filter paper. The white product was washed with 20 ml of tetrahydrofuran by centrifugation 3 times at 9000 rpm for 5 min in a Beckman Coulter C0650 fixed-angle rotor (Beckman Coulter, Brea, CA, USA). The product was vacuum-dried over night at 30 °C.

In a 100 ml 4-neck round-bottom flask, 20 ml of 1-octadecene, 0.277 g of HDA·HCl, 0.207 ml of OA, and 0.084 ml of OAm were added and magnetically stirred together. The reaction flask was connected to the Schlenk line through the center opening. A J-KEM Scientific Model Apollo temperature controller (J-KEM, St. Louis, MO, USA) was used to control and monitor the temperature of the solution with an adjacent heating mantle placed under the flask and a thermocouple placed in a second opening of the flask through a plastic septum stopper. Two rubber septum stoppers were added to the remaining two openings of the flask to allow for a closed system. The solution was vacuumed and purged with ultrapure argon under a Schlenk line 3 times and continuous flow of argon was used throughout the synthesis. The solution was then heated to 120 °C at a 15 °C/min rate to react for 30 min. The temperature was further raised to 180 °C at a 2 °C/min rate and 1.4 ml of Fe(CO)_5_ was loaded into a 3 ml syringe with a 6-inch, 18-gauge stainless steel needle. The loaded syringe was placed onto a syringe pump, and the needle was inserted directly into the reaction solution through a rubber septum stopper. The precursor was injected at a 100 μl/min rate using the syringe pump once the reaction solution reached 180 °C. After all of the precursor was dispensed, the syringe and needle were removed, and the reaction continued at 180 °C for 2 h before being cooled to room temperature. The reaction flask was removed from the setup and exposed to ambient conditions. An external magnet was placed at the base of the reaction flask and the supernatant was decanted and discarded, leaving the resultant nanoparticles on the magnetic stir bar. The nanoparticles were rinsed from the reaction flask and stir bar into a clean beaker using hexane. To separate the nanoparticles from hexane, the beaker was placed on an external magnet, allowing the particle to be immobilized while the hexane was decanted. A total volume of 10 ml of absolute ethanol was added to the beaker with the nanoparticles and sonicated for 2 min to allow for even dispersion. The particles were pipetted into centrifuge tubes and washed 3 times by centrifugation in 60 ml of absolute ethanol at 8000 rpm for 10 min. Half of the total yield was dispersed in 5 ml of chloroform and stored in the refrigerator. The other half was vacuum-dried to powder in an antechamber of an argon atmosphere glovebox for 15 min before being moved to the main glovebox chamber for storage and later characterization.

Variations in the above procedure were performed which included changing the injection rate of the precursor to 50 and 25 μl/min. In addition, different initial surfactants including HDA and ODA were tested in the replacement of OAm, OA, and HDA·HCl mixture at the 3 varying speeds. For the HDA sample, 0.024 g (0.1 mmol) of HDA was mixed with 20 ml of 1-octadecene and heated to 120 °C to react for 30 min. For the ODA sample, 0.027 g (0.1 mmol) of ODA was mixed with 20 ml of 1-octadecene and heated to 120 °C and reacted for 30 min. The subsequent steps for each sample were the exact same as mentioned previously. Each sample was named based on the surfactant used and injection rate. The first samples using oleylamine and oleic acid are named OAm/OA 100, OAm/OA 50, and OAm/OA 25. Samples that used HDA and ODA were labeled similarly.

### DSPE-mPEG coating:

A total of 2 ml of chloroform dispersed nanoparticles was mixed with 0.4 ml of DSPE-mPEG and 9.6 ml of chloroform in a 100 ml round-bottom flask. The solution was sonicated for 10 min. Then, the chloroform was evaporated with a rotary evaporator for 1 h in a 60 °C water bath under vacuum. A total volume of 4 ml of ultrapure water was added to the flask after evaporation and sonicated for 1 min to evenly disperse particles in water. The solution was transferred to Vivaspin Turbo 15 filter tubes (Sartorius) (Fisher Scientific, Waltham, MA, USA) and centrifuged for 1 h at 5000 rpm to purge excess DSPE-mPEG. The coated particles in the filter tube were pipetted into storage vials with 1–2 ml of ultrapure water to redisperse the particles in an aqueous medium. The particles were stored in a refrigerator for further cytotoxicity testing.

### Acid digestion of coated nanoparticles:

Inductively coupled plasma–optical emission spectrometry (ICP-OES) was performed on coated FeNP samples prior to cytotoxicity studies to accurately determine Fe concentrations. Preparation of samples followed the SW-846 test method 6010 and referenced previous procedures [29]. To obtain theoretical concentrations of coated nanoparticle solutions, masses of empty vials were recorded and 100 μl of dispersed samples was pipetted into separate vials. The particles were magnetically separated with an external magnet; excess water was decanted; and the particles were further vacuum-dried under argon in the vials. The final vial weight with dried nanoparticles were reweighed for each sample. The difference between the final and initial vial masses was used to calculate the mass of nanoparticles present from 100 μl of the initial solution to determine mass concentration values. Mathematical adjustments were made for each sample in order to acid digest an appropriate amount to obtain a final concentration around 50 mg/l Fe. After estimations were made, 500 μl of coated FeNPs dispersed in water were mixed with 2 ml of 1 M nitric acid and 2 ml of 37% HCl in a 25 ml volumetric flask. The solution was sonicated for 5 min before heating in an 80 °C water bath for 1 h. The solution was gravity-filtered through a Whatman filter paper with a pore size of 0.45 μm into a 200 ml volumetric flask. The initial volumetric flasks were rinsed 3–5 times with ultrapure water and poured over the filter. The final solution was brought to the appropriate 200 ml volume with ultrapure water to obtain an Fe concentration of around 50 mg/l for ICP-OES measurements.

### Characterizations:

Transmission electron microscopy, X-ray diffraction, Mössbauer spectroscopy, and vibrating sample magnetometry were performed. Due to the polydispersity observed in the samples using HDA and ODA as surfactants, only the samples using OAm/OA were tested for initial cytotoxicity studies.

### Transmission electron microscopy (TEM):

TEM was performed at the Vanderbilt Institute of Nanoscience and Engineering (VINSE), Vanderbilt University. An FEI Tecnai Osiris TEM system (Thermo Fisher Scientific, Hillsboro, OR, USA) was operated between 80 and 200 kV. A total of 100 μl of chloroform dispersed nanoparticles was added to 2 ml of chloroform. A drop of the diluted nanoparticle solution was added to carbon-coated TEM grids and allowed to dry in air before imaging. ImageJ 1.54r processing software was used to measure nanoparticle diameter, core, and shell size. An average of 200 nanoparticles were measured in order to obtain averages and standard deviations.

### X-ray diffraction (XRD):

XRD measurements were performed using a Rigaku SmartLab 3 kW X-ray Diffractometer (Rigaku, Cedar Park, TX, USA) with a Cu Kα X-ray source in a 2θ range of 20–90° at UTSI. Data was fitted using the Rigaku SmartLab software 5.0.

### Mössbauer spectroscopy:

Mössbauer Spectroscopy measurements were performed with a ^57^Co source, a Janis SHI-850–5 cryogen-free cryostat (Janis, Woburn, MA, USA), and Lakeshore 325 Temperature Controller (Lakeshore, Westerville, OH, USA). Hyperfine interaction parameters were analyzed using a least-squared fitting model with Mössbauer GenFit Software 2.0 (R. S. Preston and D. E. Brown). Powdered samples previously stored in an Ar atmosphere glovebox were loaded and sealed into Mössbauer cups for characterization under vacuum at 293 K and 6 K.

### Vibrating sample magnetometry (VSM):

VSM measurements were performed at the Institute for Advanced Materials and Manufacturing (IAMM) at the University of Tennessee Knoxville campus. A DynaCool-Advanced VSM system (Dyna-Cool, San Diego, CA, USA) using a ±4 T magnetic field at 300 K was used. The data was fitted with a modified Langevin function in OriginLab software (OriginPro 2026) and reported magnetic saturation values are based on the data fit.

### Cytotoxicity:

Cytotoxicity studies were performed at Vanderbilt University. A 4T1 murine breast cancer cell line was seeded into a 96-well plate 24 h prior to FeNP introduction. The cells were dispersed in RPMI 1640 media with 10% FBS and 1% pen/strep and stored in a 5% CO_2_ incubator to culture. DSPE-mPEG-coated FeNPs in aqueous solution were serially diluted using the previously mentioned cell culture media to obtain 100, 50, 25, and 12.5 μg Fe/ml doses. All samples were performed in triplicate for each dose. Each sample was incubated with the cells for 24 h and a Celltiter-Glo assay (Promega, Madison, WI, USA) was performed to determine cell viability. Results were compared to untreated 4T1 cells as a control.

## 3. Results

TEM results of all samples are shown in Figure 2 at 100 nm and 20 nm scales, and elemental mapping of all samples are shown in Figure 3 to identify and assess core–shell structures and composition. A summary of the diameter, core and shell dimensions for all samples at all injection rates are shown in Table 1. Average diameter, core, and shell dimensions for the HDA 25 and ODA 25 samples are not included in Table 1. For these two samples, the nanoparticles began to merge into larger masses up to 60 nm in size and distinction of individual nanoparticle morphologies was difficult to observe. From these results, all OAm/OA samples at all injection rates of 100, 50 and 25 μl/min were monodispersed. The OAm/OA samples appeared largely spherical and consisted of an iron core and iron oxide shell. However, the iron core began to decrease as the injection speed decreased. In addition, at the lowest injection rate of25 μl/min, a separation between the core and shell of the nanoparticles began to form. This could be due to the longer reaction time and is consistent with the Kirkendall effect [30].

For the samples using HDA, as injection rate decreased, the overall size of particles began to increase. Generally, all HDA samples possessed an iron core and iron oxide shell. However, an increase in polydispersity and agglomeration of the samples occurred as injection rate decreased. The HDA 100 sample was observed to have less flocculation in solution, but the shape of the nanoparticles began to become more prolate compared to spherical nanoparticles that were noted in the OAm/OA samples.

Similar results were observed in the ODA samples when compared to the HDA samples at all injection speeds. Generally, size increased as injection rate decreased. The ODA 100 and ODA 50 samples had relatively good dispersibility from the images, but the nanoparticle shapes were irregular. In addition, as the injection rate decreased, polydispersity of nanoparticles began to increase. Similarly to other samples, the ODA samples consisted of an iron core and iron oxide shell.

From the XRD spectra (Figure 4), the OAm/OA 100, 50 and 25 samples all displayed good crystallinity with peaks indicative of α-Fe at 45°, 65°, and 82°. From the HDA samples, all injection speeds except for 25 μl/min displayed broad α-Fe peaks at 45° and 65°, suggesting amorphous or polycrystalline properties. From the ODA samples, the ODA 100 sample had a stronger amorphous or polycrystalline peak at 45° compared to the ODA 50 and ODA 25 samples. The ODA 50 and ODA 25 samples displayed little to no crystalline peaks, suggesting the samples were amorphous.

Stacked Mössbauer spectra of all samples measured at 293 K and 6 K are shown in Figure 5. Corresponding fitting results for each sample from 293 K measurements are displayed in Table 2 and fitting results from 6 K measurements are displayed in Table 3. From room temperature measurements, all samples were relatively similar consisting of two components, a red sextet and a green doublet. For the OAm/OA 100, OAm/OA 50, and OAm/OA 25 samples, the first red component had a more defined sextet pattern with a hyperfine value around 32 T, and isomer shift and quadrupole splitting of 0 mm/s. This component is attributed to α-Fe, which is present in the core of the nanoparticles from TEM imaging. For all HDA and ODA samples, the first component consisted of a broad red sextet with a hyperfine value between 21.9 and 23.3 T, isomer shift between 0.13 and 0.25 mm/s, and quadrupole splitting between 0.03 and 0.12 mm/s, which is indicative of amorphous iron [31]. The second component for all samples consisted of a green doublet pattern that had an isomer shift between 0.32 and 0.43 mm/s and quadrupole splitting between 0.80 and 1.01 mm/s. Upon initial fitting, this doublet pattern is indicative of γ-Fe_2_O_3_ [32]. However, low temperature measurements were performed to further confirm this iron oxide component.

For the OAm/OA samples at all injection speeds, the low temperature spectra were similar. Each sample consisted of a defined red sextet pattern that had a hyperfine field around 34 T, isomer shift between 0.08 and 0.12 mm/s, and quadrupole splitting of 0 mm/s, which is indicative of α-Fe. The second component consisted of a green sextet that had a hyperfine field between 48 and 49 T, isomer shift between 0.42 and 0.54 mm/s, and quadrupole splitting between 0.11 and 0.15 mm/s, which is indicative of A sites forγ-Fe2O3. The third component consisted of a blue sextet that had a hyperfine field between 47.3 and 48.6 T, isomer shift between 0.19 and 0.54 mm/s, and quadrupole splitting between −0.16 and −0.58 mm/s, which is indicative of B sites for γ-Fe_2_O_3_. From low temperature measurements, superparamagnetic characteristics were observed for the γ-Fe_2_O_3_ component due to it splitting from a doublet pattern at 293 K measurements to sextet patterns at 6 K [29].

For the HDA and ODA samples at all injection rates, all spectra looked similar at 6 K. They all consisted of a sextet pattern (shown in red) with a hyperfine field between 22.6 and 25.1 T, an isomer shift between 0.23 and 0.36 mm/s, and quadrupole splitting between 0 and 0.05 mm/s, which is indicative of amorphous iron. The second component consisted of a sextet pattern (shown in green) that had a hyperfine field between 48.5 and 49.4 T, isomer shift between 0.39 and 0.53 mm/s, and quadrupole splitting between 0.10 and 0.25 mm/s, which is indicative of A sites for γ-Fe_2_O_3_. The third component consisted of a blue sextet with a hyperfine field between 47.5 and 48.5 T, isomer shift between 0.20 and 0.47 mm/s, and quadrupole splitting between −0.11 and −0.51 mm/s, which is indicative of B sites for γ-Fe_2_O_3_ [33, 34]. From the low temperature spectra, the doublet component (shown in green) is attributed to γ-Fe_2_O_3_; the ODA and HDA samples displayed superparamagnetic characteristics due the spectrum splitting into sextet patterns at low temperatures.

Magnetic hysteresis (MH) curves of all samples are shown in Figure 6 and magnetic saturation values for all samples are listed in Table 4. All MH curves were fitted with a modified Langevin function using Origin Lab processing software. From the results, all of the OAm/OA samples displayed superparamagnetic characteristics. The OAm/OA 100 sample had the highest magnetic saturation at approximately 138 emu/g. Magnetic saturation decreased as the injection rate decreased for the OAm/OA 50 and OAm/OA 25 samples, with OAm/OA 25 having a significant drop in magnetic saturation of 46 emu/g compared to the other OAm/OA samples being above 100 emu/g. The HDA and ODA samples also displayed superparamagnetic characteristics and had relatively similar magnetic saturation values between 30 and 45 emu/g. The ODA 25 sample had the lowest magnetic saturation value of 12 emu/g.

Cytotoxicity studies were only performed on the OAm/OA samples since the HDA and ODA samples agglomerated and had low dispersibility. For all OAm/OA samples, four doses of FeNPs were tested: 100, 50, 25, and 12.5 μg Fe/ml. Each dose was incubated with 4T1 cells for 24 h, and a Cell-Titer Glo Assay was performed to determine overall cell viability. Results of these initial tests are shown in Figure 7. All OAm/OA samples displayed good cell viability for all doses and an increase in cell viability as Fe concentration decreased. The OAm/OA 100 sample had slightly less cell viability compared to the OAm/OA 50 and OAm/OA 25 samples at all doses, with the 100 μg Fe/ml dose being around 73% for cell viability for the OAm/OA 100 sample. In addition, for the OAm/OA100 sample, the 50 μg Fe/ml and 25 μg Fe/ml doses had around 82% cell viability and the 12.5 μg Fe/ml dose had around 99% cell viability.

Cell samples that were not treated with FeNPs were used as controls and established the 100% viability baseline. Although all samples were incubated for identical durations, we note that some measures suggest greater than 100% viability. However, the results within each coating type are self-consistent among FeNP concentrations. Absolute cytotoxicity of the 12.5 μg/ml Fe treatment was negligible (for all coatings) based on the quantitative CellTiterGlo response. Relative to the 12.5 μg/ml treatments, our results suggest reduced viability as a function of FeNP dose for each coating type and a trend for the highest viability among the 25 OA treatments and lowest viability among the 100 OA treatments.

The OAm/OA 50 and OAm/OA 25 samples displayed cell viability above 100% for all doses with the exception of the 100 μg/ml dose for the OAm/OA 25 sample. A potential reason for these higher cell viability percentages could be due to the PEG coating on the sample. Previous studies have shown PEG to cause some cell proliferation effects [35]. However, considering the OAm/OA 100 sample did not display this phenomenon, further tests would be needed to confirm the cause of this observation. In general, good cell viability for all three samples was observed.

We also note that the FeNP doses used were significantly greater than those used clinically for enhanced MR imaging using Feridex, an iron oxide-based contrast agent. At a Feridex dose of 0.56 mg/kg in a 65 kg human, the 100 μg/ml concentration in the 96-well plate is 16 million-fold greater (based on mg/kg) and is approximately 7000-fold greater based only on the mass of cells that line the human vasculature. Thus, the doses applied in our cytotoxicity study were considerably greater than the FeNP exposure that can be reasonably presumed in vivo.

## 4. Discussion

From testing various injection rates of iron pentacarbonyl along with different types of surfactants, overall particle size increased as the injection rate of the iron precursor decreased for samples using HDA and ODA as surfactants. Little [36] difference in size variations for samples using OAm/OA was observed when injection rate varied. Previous studies utilizing OAm and OA as surfactants have found that particle sizes can only reach around 14–15 nm in total diameter [37]. Compared to OAm and OA, surfactants such as HDA and ODA lack C=C bonds that strongly bind to nanoparticles during growth, which can allow for larger variation in nanoparticle sizes. Due to this lack of C=C bonds, weak interactions between the growing nuclei surface and surfactant causes rapid growth to allow for larger sizes. However, due to this rapid growth, amorphous or polycrystalline characteristics are common for the formed nanoparticles, which was confirmed from XRD measurements [38, 39]. From TEM results, larger nanoparticle sizes were achieved with HDA and ODA surfactants as injection rates decreased. Although the nanoparticles maintained an iron core–iron oxide shell structure, the nanoparticles were polydisperse, which is common when utilizing a LaMer growth mechanism [26]. In order to prevent the polydispersity, either a higher concentration of precursor or a slower injection rate of the precursor could be used to allow for a continuous growth of uniform nanoparticles. This would allow full extension of the reaction to reach a supersaturation state [26]. In addition, a higher amount of surfactant could be tested, since only 0.1 mmol of the HDA and ODA was used for their respective syntheses. Due to the low volume of surfactants used, the ligand amount in the system may be insufficient to provide proper steric hindrances as the nuclei grow, causing a higher agglomeration rate.

From Mössbauer characterization, all samples had similar spectra for room temperature measurements, consisting of two components indicative of α-Fe and γ-Fe_2_O_3_. The OAm/OA samples at all injection speeds displayed a very distinct sextet indicative of α-Fe compared to the HDA and ODA samples, which had an amorphous Fe component at all injection speeds. From low temperature measurements, all OAm/OA samples had similar spectra indicative of α-Fe and γ-Fe_2_O_3_. All HDA and ODA samples had similar Mössbauer spectra from low temperature measurements consisting of three components indicative of amorphous Fe andA and B sites for γ-Fe_2_O_3_. In addition, all samples had a superparamagnetic iron oxide component due to the doublet pattern from room temperature measurements splitting into sextet patterns when measured at low temperature.

From magnetization measurements, all samples displayed superparamagnetic qualities due to the little to no presence of hysteretic remanence. The magnetic saturation for the OAm/OA samples decreased as the injection rate decreased. From TEM imaging, it was observed that a hollow or yolk-like morphology began to form as injection rate decreased for the OAm/OA samples, causing a loss of iron material between the core and the shell.

This phenomenon is due to the nanoscale Kirkendall effect [30]. The Kirkendall effect is when the outward diffusion of cations through the shell is faster than the inward diffusion of anions. This variation in diffusion rate causes vacancies in the metal core that coalesce into single or multiple pores that consume the core and form hollow structures [40]. This hollowness between the core and shell could be attributed to the lower magnetic saturation for these samples. In addition, a higher percent of iron oxide was present as the injection rate decreased for the OAm/OA samples, which can also cause lower magnetic saturation. For all HDA and ODA samples, magnetic saturation values were similar between 30 and 45 emu/g. The ODA 25 sample had the lowest magnetic saturation out of all samples of around 12 emu/g. The low values for these samples compared to the OAm/OA samples is most likely due to the amorphous or polycrystalline nature, which was confirmed from XRD measurements. Previous studies that used similar surfactants with amine functional groups have reported similar magnetic saturation values and amorphous characteristics as well [41]. Due to the disordered crystal structure and randomly coupled electrons, lower magnetic properties are observed.

Finally, from cytotoxicity tests on the OAm/OA samples, all samples displayed relatively good cell viability when compared to untreated control samples. The OAm/OA 100 sample had slightly lower cell viability compared to the OAm/OA 50 and OAm/OA 25 samples but still had good cell viability at 25 and 12.5 μg/ml doses. Some cell proliferation appeared to occur for the OAm/OA 50 and OAm/OA 25 sample, which could be attributed to the PEG coating on the sample [35, 36]. However, this phenomenon did not occur for the OAm/OA 100 sample that was also PEG-coated, suggesting that further testing is needed to confirm the reason for this observation [42].

## 5. Conclusions

A continuous injection of iron pentacarbonyl at speeds of 100, 50, and 25 μl/min was investigated in order to form larger FeNPs. In addition, at each injection speed, three different surfactants were used to see if the surfactant type would also affect the size and composition of the nanoparticles. It was found that with samples synthesized using OAm/OA as the surfactant, average particle size remained the same at around 14 nm regardless of injection rate. However, as the injection rate decreased, the particles began to form a yolk-like structure where a separation between the core and shell occurred due to Oswald ripening and the Kirkendall effect. Samples using HDA and ODA produced larger particles at the highest injection speed of 100 μl/min. However, the particles had an irregular shape. At the lower injection speeds of 50 and 25 μl/min, samples using HDA or ODA as the surfactants agglomerated and formed polydisperse nanoparticles. For the samples that did not agglomerate, the nanoparticles consisted of an iron core and γ-Fe_2_O_3_ shell. The OAm/OA samples displayed good crystallinity at all injection speeds However, the HDA and ODA samples show polycrystalline or amorphous characteristics at all injection speeds.

From magnetization tests, the OAm/OA 100 sample had the highest magnetic saturation of around 138 emu/g. The HDA and ODA samples had lower magnetic saturation values between 30 and 45 emu/g, at all injection rates, which is attributed to the polycrystalline or amorphous nature of the nanoparticles. Furthermore, from cytotoxicity studies, samples using OAm/OA displayed low cytotoxicity at all injection rates.

We conclude that this method has the potential to result in larger nanoparticles. The HDA 100 and ODA 100 samples may have potential for MPI applications due to the targeted size and composition being obtained. In order to make the nanoparticles optimal for MPI applications, further work is needed to develop larger, monodisperse nanoparticles with good crystallinity.

For the HDA and ODA samples, it was clear that larger particles could be produced at lower injection speeds; however, the particles agglomerated and became polydisperse. To mitigate agglomeration and lessen polydispersity, a slower injection rate of the precursor could be implemented, and an increased amount of surfactant could be used.

## Figures and Tables

**Figure 1 •:**
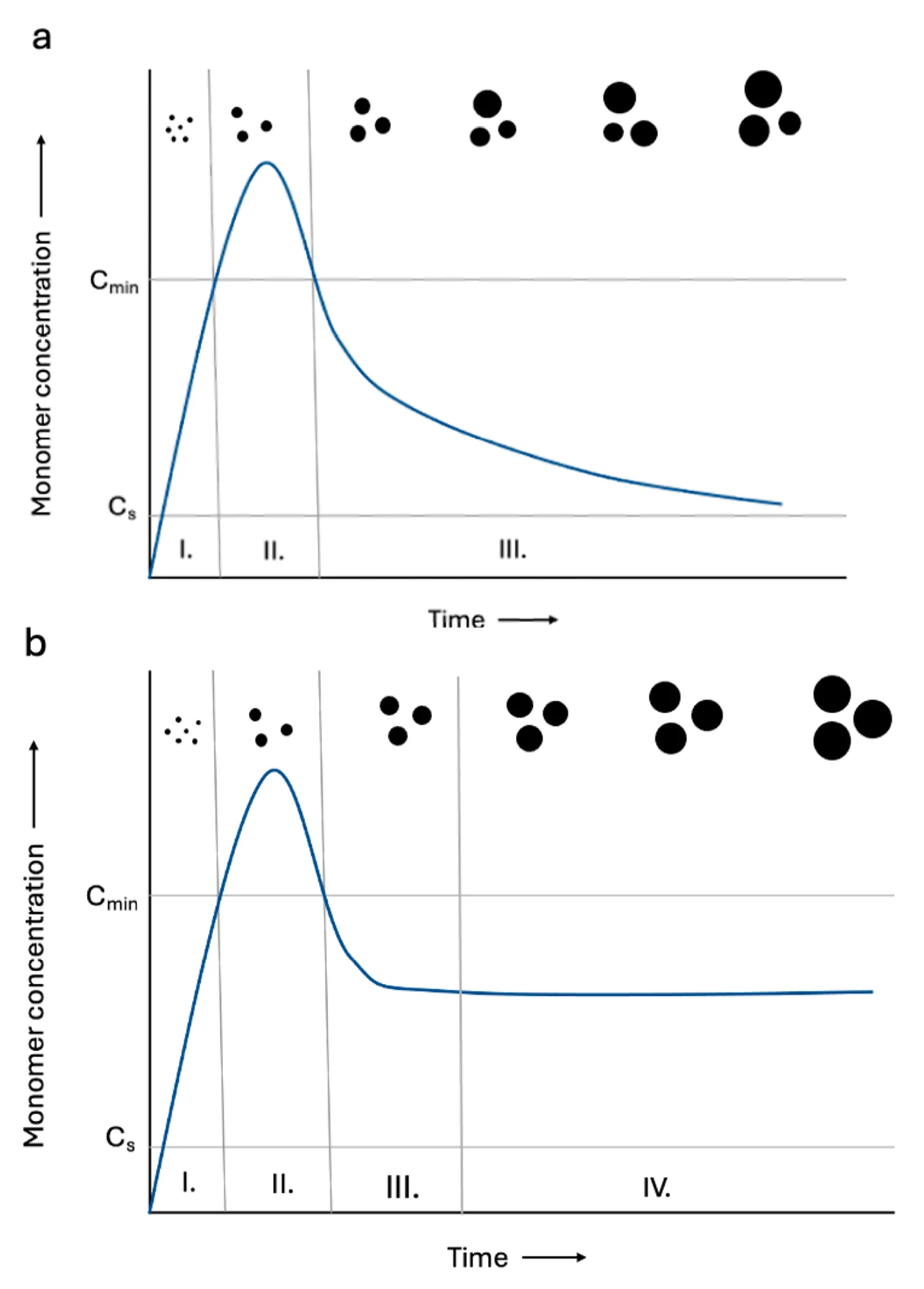
(**a**) Original LaMer mechanism: comprised of three stages as labeled by the numerals I-III. Stage I is the precursor monomer concentration increasing to the critical supersaturation concentration to initiate nucleation (Cmin). In stage II, the precursor monomer concentration decreasing from supersaturation. Finally in stage III, the precursor monomer concentration decreases below saturation levels required for nucleation, causing the nanoparticles to grow by adding monomer to existing particle surfaces. Once precursor monomer concentration is close to lower solubility limit (CS), growth continues via Ostwald ripening which leads to nanoparticle polydispersity as shown by the black dots. (**b**) Extended LaMer mechanism for particle growth over time. It follows the original mechanism through stages I and II, but stage III does not decrease towards CS but instead stabilizes at a steady state concentration. This adds a new stage of growth in stage IV, where a steady state precursor monomer concentration continues stable nanoparticle growth without any Ostwald ripening—which results in larger particle sizes with narrower size dispersion as shown by the black dots.

**Figure 2 •:**
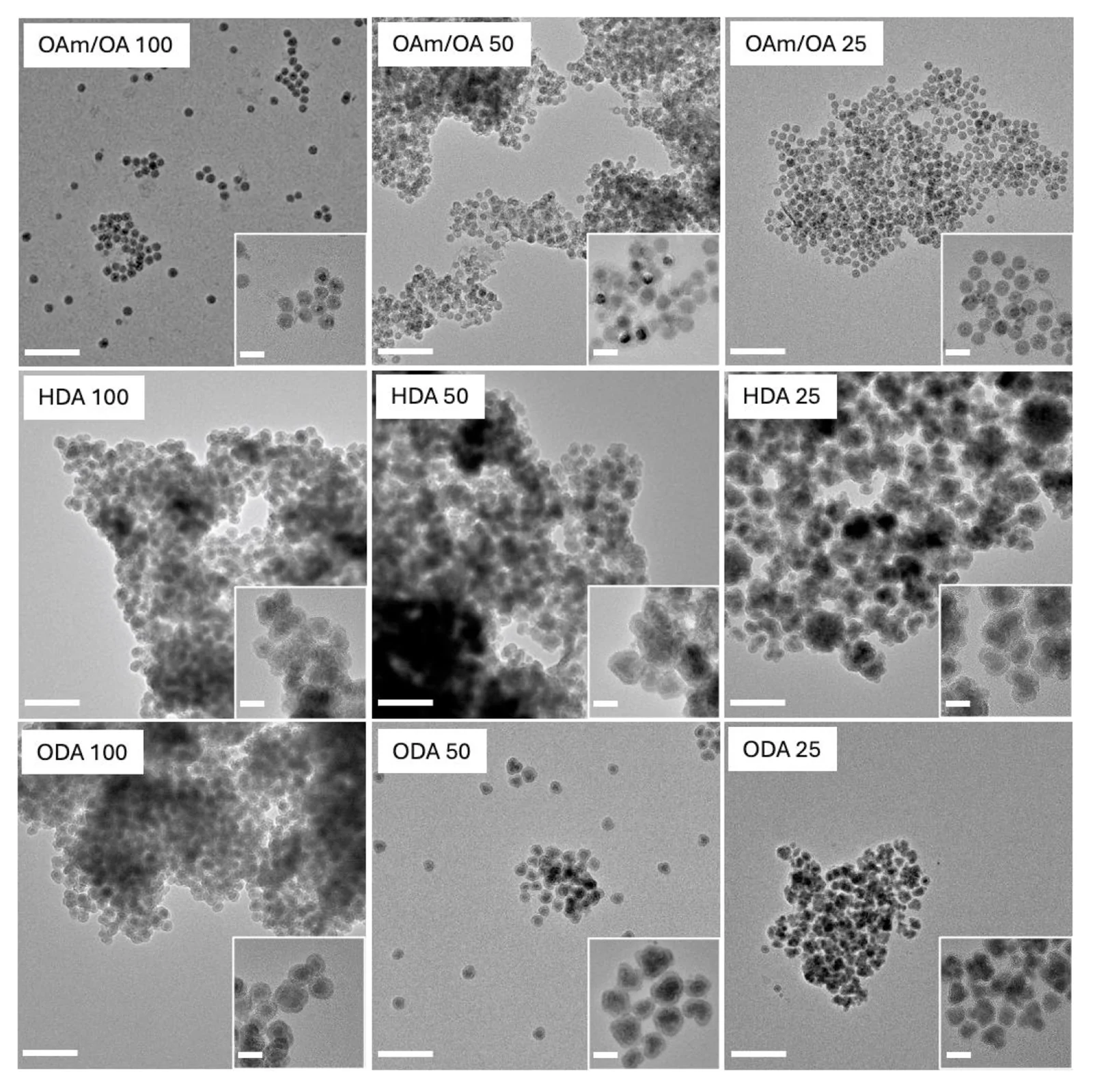
TEM image of all samples using a continuous injection of iron precursor. Larger scale bar: 100 nm. Inset image scale bar: 20 nm. Surfactants and injection rates (μl/min) are identified in the upper left side label.

**Figure 3 •:**
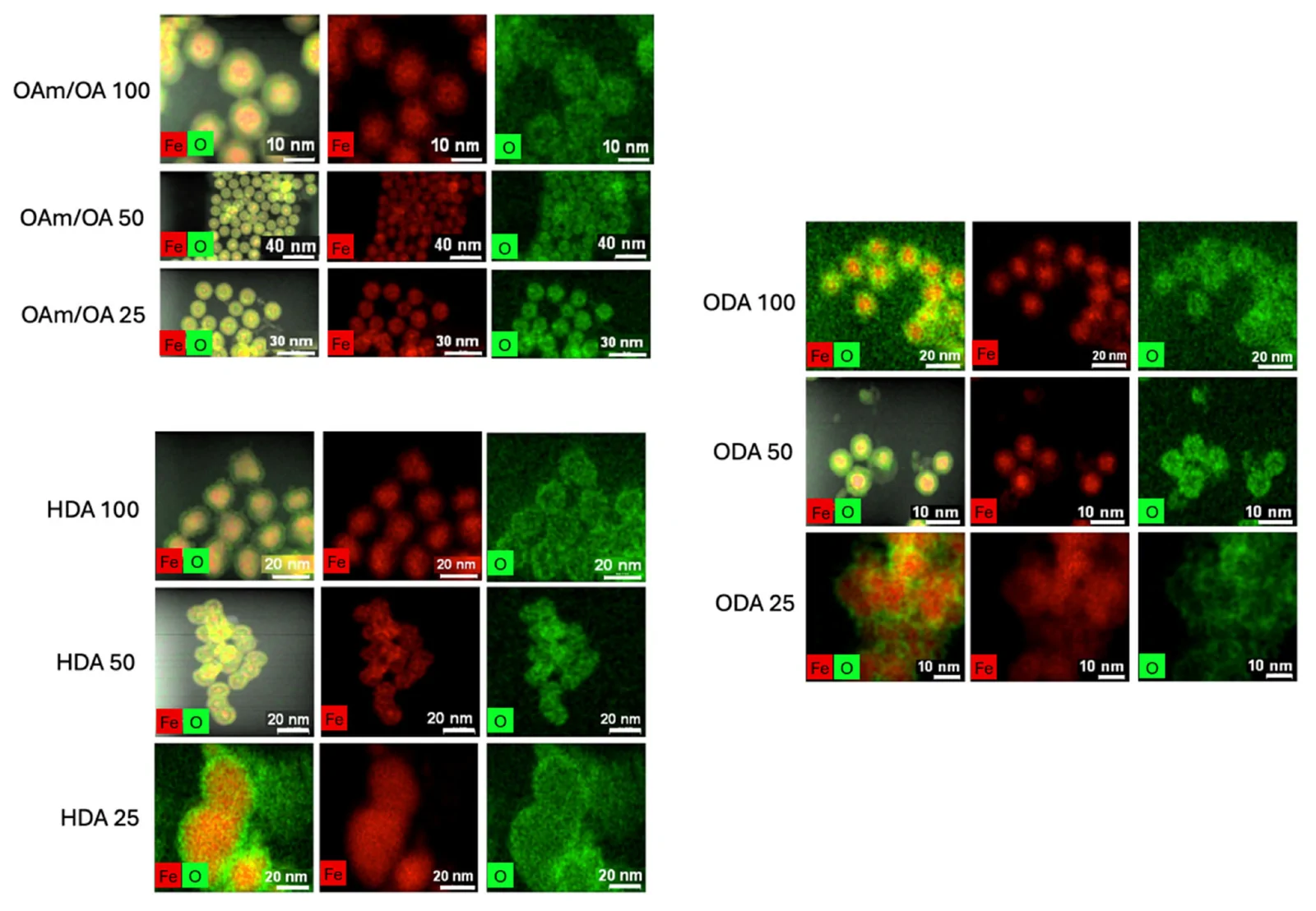
Elemental mapping of all samples to display iron core–iron oxide shell structures. Elemental iron is shown in red, and oxygen is shown in green. Composition and injection rates (μl/min) are displayed on the left of each image set. All images taken in High-Angle Annular Dark Field (HAADF) mode.

**Figure 4 •:**
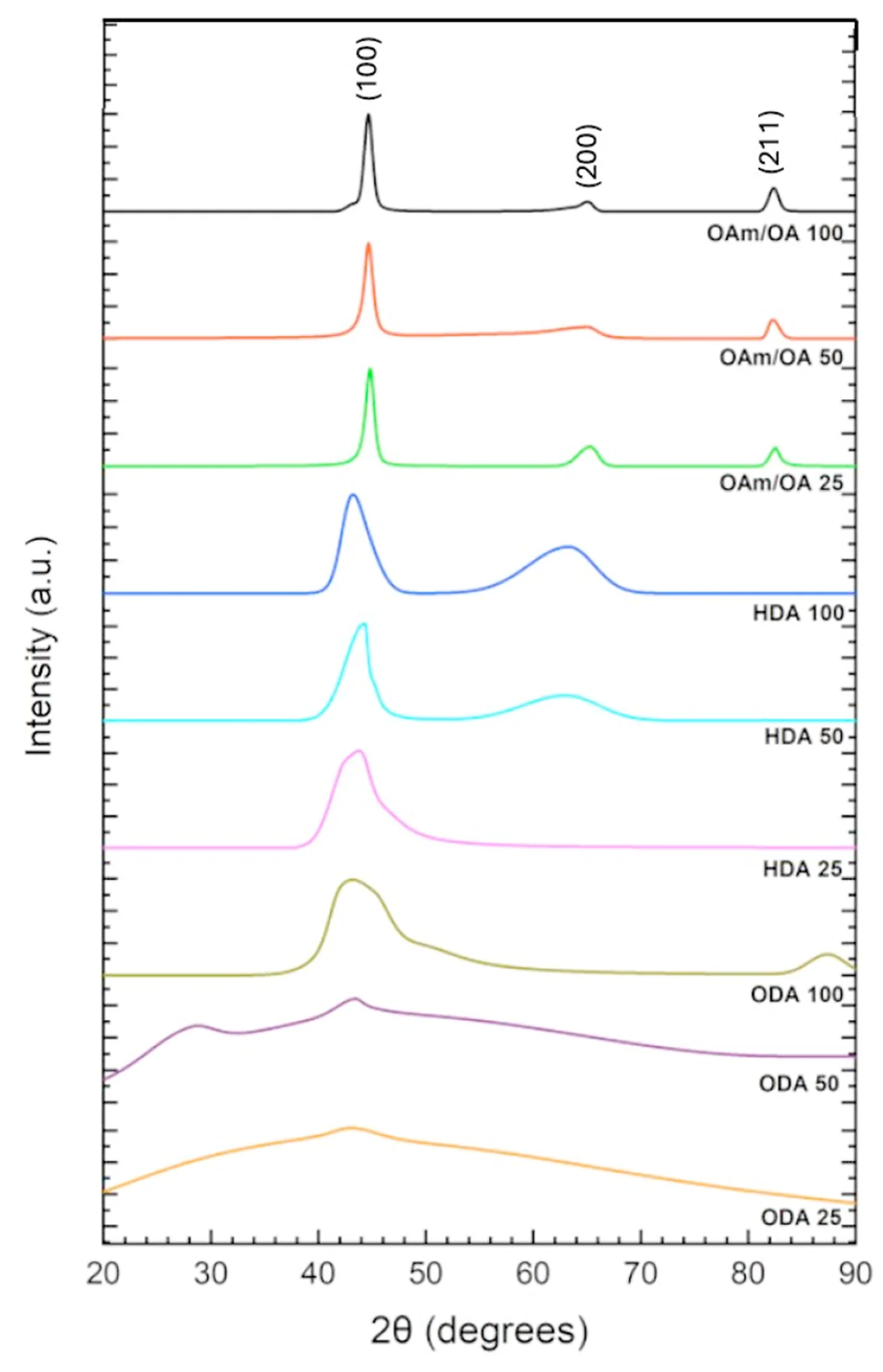
Stacked XRD spectra of all samples: OAm/OA, 100, 50, 25; HDA, 100, 50, 25; and ODA, 100, 50, and 25.

**Figure 5 •:**
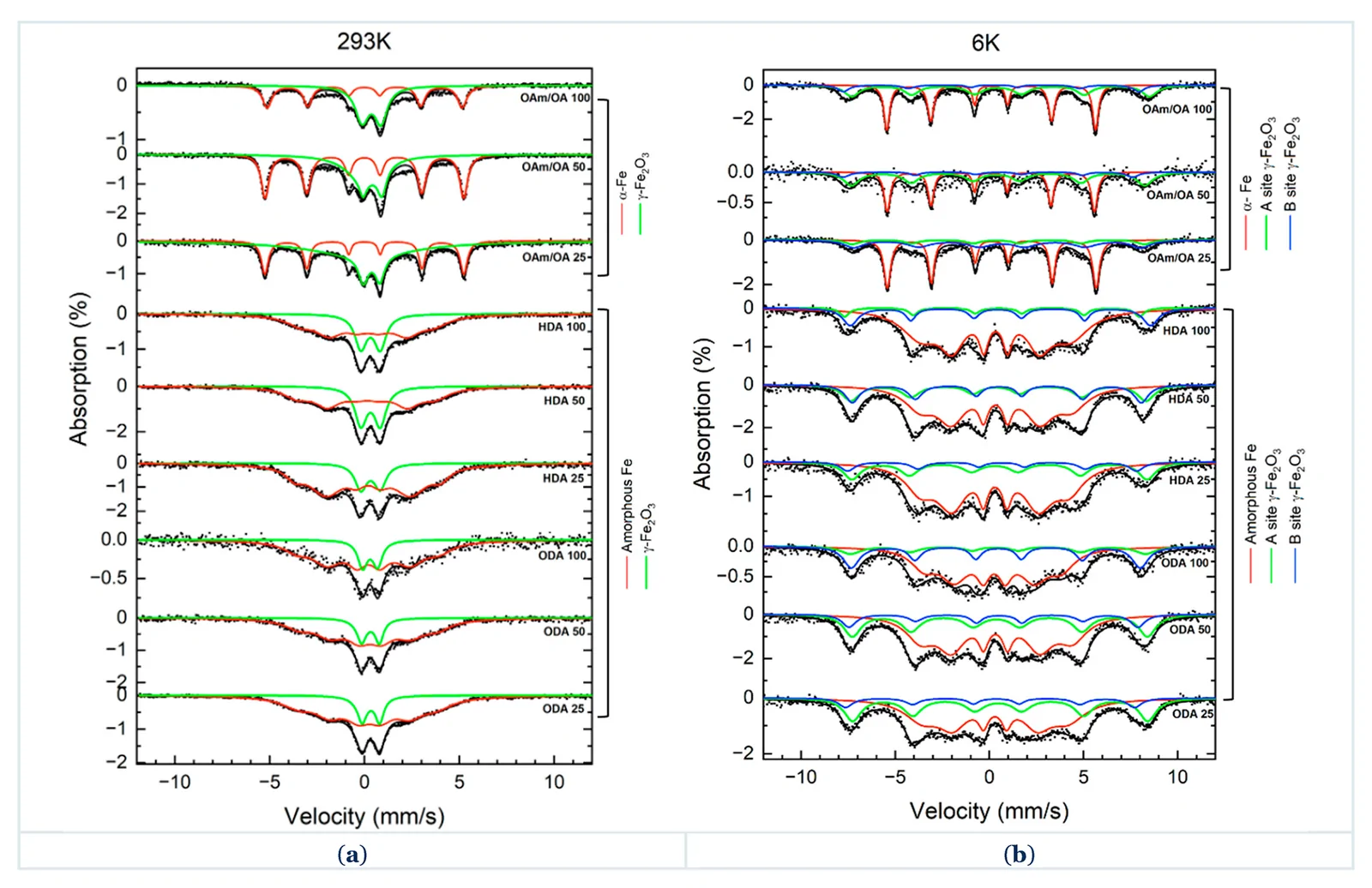
Stacked ^57^Fe Mössbauer spectra of all samples using a continuous injection method at 293 K (**a**) and 6 K (**b**).

**Figure 6 •:**
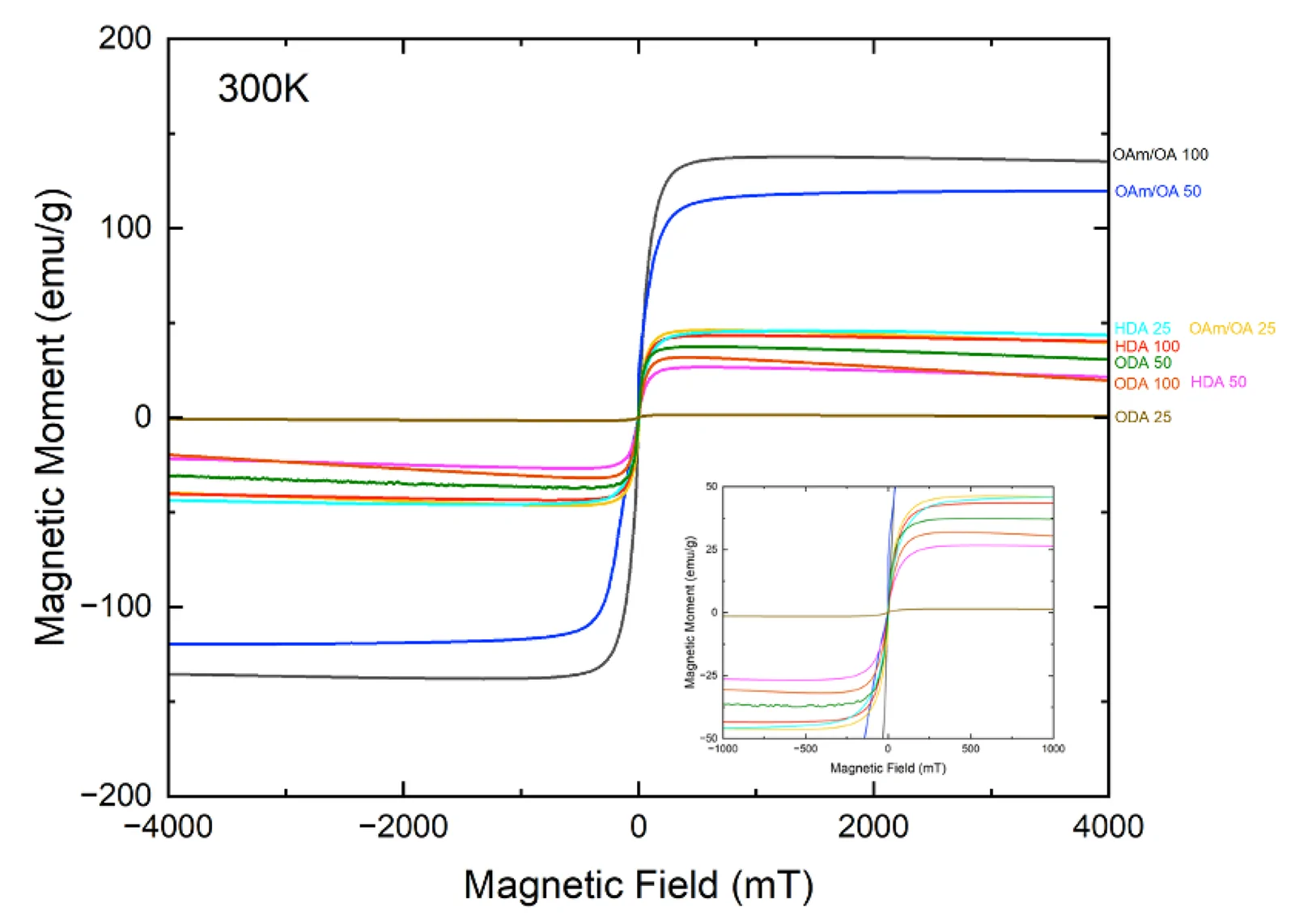
MH curves of all samples measured at 300 K.

**Figure 7 •:**
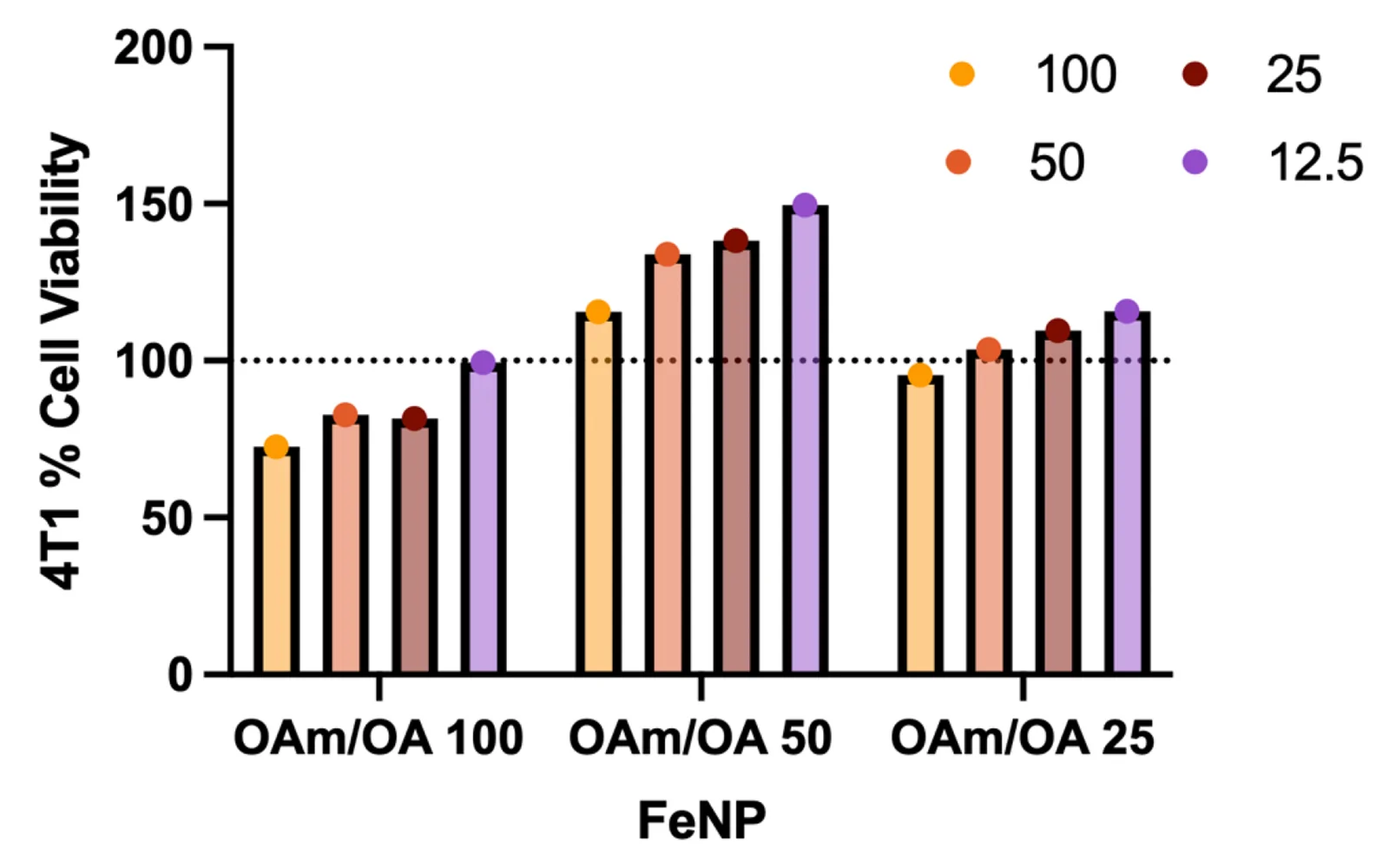
Cell viability results of OAm/OA 100, OAm/OA 50, and OAm/OA 25 samples at 100, 50, 25, and 12.5 μg Fe/ml doses.

**Table 1 •:** Summary of diameter, core, and shell measurements for all samples at all injection rates. An average of 200 nanoparticles were counted to obtain averages and standard deviations. Results are in nm and presented as mean ± std. dev. Due to large mass formations, diameter, core, and shell measurements for the HDA 25 and ODA 25 are not included.

Sample	Diameter	Core	Shell
OAm/OA 100	15.7 ± 1.6	5.7 ± 1.0	5.4 ± 0.7
OAm/OA 50	15.5 ± 1.5	5.4 ± 0.7	5.0 ± 0.8
OAm/OA 25	14.8 ± 0.9	4.9 ± 0.7	5.1 ± 0.5
HDA 100	22.0 ± 3.2	13.8 ± 2.4	4.0 ± 1.2
HDA 50	24.3 ± 3.8	14.3 ± 3.9	5.1 ± 0.8
HDA 25	Polydisperse/Large Masses Formed		
ODA 100	22.4 ± 2.4	12.4 ± 2.3	5.4 ± 1.0
ODA 50	26.6 ± 4.6	14.4 ± 3.7	5.3 ± 1.1
ODA 25	Polydisperse/Large Masses Formed		

**Table 2 •:** Values of hyperfine parameters for all samples using a continuous injection method at 293 K. *B*_hf_ is the hyperfine magnetic field, δ is the isomer shift, ∆*E_Q_* is the quadrupole splitting, and Γ is the linewidth (FWHM). Typical errors are ±0.5 T for *B*_hf_, ±0.05 mm/s for δ, ±0.02 mm/s for ∆*E_Q_*, and ±0.05 mm/s for Γ and ±3% for relative area.

Sample	Component	*B_hf_* (T)	δ (mm/s)	∆*E_Q_* (mm/s)	Γ (mm/s)	Relative area (%)	Attribution
OAm/OA 100	1	32.0	0.00	0.00	0.39	48	α-Fe
	2	0.00	0.38	0.90	0.80	52	γ-Fe_2_O_3_
OAm/OA 50	1	32.5	0.00	0.00	0.46	56	α-Fe
	2	0.00	0.43	1.00	0.60	44	γ-Fe_2_O_3_
OAm/OA 25	1	32.5	0.00	0.00	0.30	31	α-Fe
	2	0.00	0.38	0.87	0.60	69	γ-Fe_2_O_3_
HDA 100	1	21.9	0.17	0.03	1.58	71	Amorphous Fe
	2	0.00	0.36	1.01	0.75	29	γ-Fe_2_O_3_
HDA 50	1	22.5	0.13	0.05	1.72	67	Amorphous Fe
	2	0.00	0.33	1.01	0.75	33	γ-Fe_2_O_3_
HDA 25	1	23.8	0.20	0.02	1.13	82	Amorphous Fe
	2	0.00	0.36	1.01	0.75	18	γ-Fe_2_O_3_
ODA 100	1	23.3	0.20	0.03	1.16	80	Amorphous Fe
	2	0.00	0.30	0.80	0.66	20	γ-Fe_2_O_3_
ODA 50	1	22.7	0.25	0.11	1.51	82	Amorphous Fe
	2	0.00	0.32	0.93	0.89	18	γ-Fe_2_O_3_
ODA 25	1	22.6	0.21	0.12	1.59	83	Amorphous Fe
	2	0.00	0.34	0.92	0.53	17	γ-Fe_2_O_3_

**Table 3 •:** Values of hyperfine parameters for all samples using a continuous injection method at 6 K. *B*_hf_ is the hyperfine magnetic field, δ is the isomer shift, ∆*E_Q_* is the quadrupole splitting, and Γ is the linewidth (FWHM). Typical errors are ±0.5 T for *B*_hf_, ±0.05 mm/s for δ, ±0.02 mm/s for ∆*E_Q_*, and ±0.05 mm/s for Γ and ±3% for relative area.

Sample	Component	*B_hf_* (T)	δ (mm/s)	∆*E_Q_* (mm/s)	Γ (mm/s)	Relative area (%)	Attribution
	1	34.3	0.10	0.00	0.28	48	α-Fe
OAm/OA 100	2	49.0	0.54	0.15	1.06	40	A site γ-Fe_2_O_3_
	3	48.6	0.19	−0.16	0.51	12	B site γ-Fe_2_O_3_
	1	34.1	0.08	0.00	0.25	45	α-Fe
OAm/OA 50	2	48.3	0.42	0.11	1.01	45	A site γ-Fe_2_O_3_
	3	47.6	0.22	−0.58	0.70	10	B site γ-Fe_2_O_3_
	1	34.4	0.12	0.00	0.31	52	α-Fe
OAm/OA 25	2	48.0	0.42	0.11	0.88	12	A site γ-Fe_2_O_3_
	3	47.3	0.54	−0.24	1.55	36	B site γ-Fe_2_O_3_
	1	24.6	0.34	0.00	0.60	76	Amorphous Fe
HDA 100	2	49.4	0.53	0.15	0.70	17	A site γ-Fe_2_O_3_
	3	48.5	0.33	−0.35	0.55	7	B site γ-Fe_2_O_3_
	1	25.1	0.35	0.02	0.60	61	Amorphous Fe
HDA 50	2	48.5	0.43	0.11	1.00	20	A site γ-Fe_2_O_3_
	3	47.5	0.47	−0.11	0.71	19	B site γ-Fe_2_O_3_
	1	24.5	0.31	0.00	0.58	70	Amorphous Fe
HDA 25	2	48.5	0.43	0.25	1.20	22	A site γ-Fe_2_O_3_
	3	47.5	0.41	−0.51	0.71	8	B site γ-Fe_2_O_3_
	1	22.6	0.23	0.03	0.85	63	Amorphous Fe
ODA 100	2	48.5	0.39	0.15	1.00	13	A site γ-Fe_2_O_3_
	3	47.6	0.42	−0.15	0.71	24	B site γ-Fe_2_O_3_
	1	25.0	0.36	0.05	0.64	59	Amorphous Fe
ODA 50	2	48.5	0.48	0.18	1.21	29	A site γ-Fe_2_O_3_
	3	47.5	0.38	−0.31	0.71	12	B site γ-Fe_2_O_3_
	1	24.0	0.31	0.00	0.63	58	Amorphous Fe
ODA 25	2	48.5	0.53	0.10	1.21	32	A site γ-Fe_2_O_3_
	3	47.5	0.20	−0.33	0.71	10	B site γ-Fe_2_O_3_

**Table 4 •:** Summary of magnetic saturation values for all samples measured at 300 K based on fitted data.

Sample	Magnetic saturation (emu/g)
OAm/OA 100	137.9
OAm/OA 50	117.0
OAm/OA 25	46.0
HDA 100	43.4
HDA 50	26.7
HDA 25	45.9
ODA 100	31.8
ODA 50	37.5
ODA 25	12.2

## Data Availability

The data supporting the findings of this publication can be made available upon request.
